# Prediction of the binding interface between monoclonal antibody m102.4 and Nipah attachment glycoprotein using structure-guided alanine scanning and computational docking

**DOI:** 10.1038/s41598-020-75056-y

**Published:** 2020-10-26

**Authors:** Phanthakarn Tit-oon, Kannan Tharakaraman, Charlermchai Artpradit, Abhinav Godavarthi, Pareenart Sungkeeree, Varun Sasisekharan, Jarunee Kerdwong, Nathaniel Loren Miller, Bhuvna Mahajan, Amnart Khongmanee, Mathuros Ruchirawat, Ram Sasisekharan, Mayuree Fuangthong

**Affiliations:** 1 Translational Research Unit, Chulabhorn Research Institute, Bangkok, 10210 Thailand; 2 Koch Institute for Integrative Cancer Research, Massachusetts Institute of Technology, Cambridge, MA 02139 USA; 3 Department of Biological Engineering, Massachusetts Institute of Technology, Cambridge, MA 02139 USA; 4 Harvard-MIT Division of Health Sciences and Technology, Massachusetts Institute of Technology, Cambridge, MA 02139 USA; 5 Yale University, New Haven, CT 06520 USA

**Keywords:** Viral proteins, Molecular modelling

## Abstract

Nipah Virus (NiV) has been designated as a priority disease with an urgent need for therapeutic development by World Health Organization. The monoclonal antibody m102.4 binds to the immunodominant NiV receptor-binding glycoprotein (GP), and potently neutralizes NiV, indicating its potential as a therapeutic agent. Although the co-crystal structure of m102.3, an m102.4 derivative, in complex with the GP of the related Hendra Virus (HeV) has been solved, the structural interaction between m102.4 and NiV is uncharacterized. Herein, we used structure-guided alanine-scanning mutagenesis to map the functional epitope and paratope residues that govern the antigen–antibody interaction. Our results revealed that the binding of m102.4 is mediated predominantly by two residues in the HCDR3 region, which is unusually small for an antibody-antigen interaction. We performed computational docking to generate a structural model of m102.4-NiV interaction. Our model indicates that m102.4 targets the common hydrophobic central cavity and a hydrophilic rim on the GP, as observed for the m102.3-HeV co-crystal, albeit with Fv orientation differences. In summary, our study provides insight into the m102.4-NiV interaction, demonstrating that structure-guided alanine-scanning and computational modeling can serve as the starting point for additional antibody reengineering (e.g. affinity maturation) to generate potential therapeutic candidates.

## Introduction

The Nipah Virus (NiV) is a negative-sense RNA virus in the genus *Henipavirus* (along with the Hendra (HeV) and Cedar (CeV) viruses) first documented during a devastating outbreak in Malaysia in 1999^1^. Since then, the virus has caused near annual outbreaks with case fatality rate of 40–75%^2^. Human-to-human transmission has been documented in multiple outbreaks since 2004^3–5^. Outbreaks have also occurred in distribution facilities in major ports, such as a slaughterhouse in Singapore^6^. Recently in May 2018, an outbreak occurred in Southern India that was significant for substantial nosocomial transmission and an 89% mortality rate^7^. In June 2019, the same region was placed under surveillance again for fears of yet another outbreak^8^.

NiV is most commonly transmitted from fruit bats, in which it has been detected in at least 7 species of bats through Asia^9–11^, Australia^12^, Madagascar^13^, and Western Africa^14^. Further, NiV has been found to easily infect several other animal species because its cell surface receptor, Ephrin-B2/3 (EFNB2/3)—is highly conserved among orthologs^15^. The combination of significant receptor conservation, broad geographic host range, and NiV’s error-prone RNA polymerase renders NiV a tremendous public health threat with pandemic potential. Indeed, unpredictability, potential for large-scale outbreaks, and propensity for developing resistance have led Nipah and Henipaviral diseases to be deemed as one of 10 Blueprint Priority Diseases in urgent need of R&D by the World Health Organization^16^.

The dire situation faced by patients and their communities during NiV outbreaks demands rapid responses from the government and healthcare industries. No approved vaccines or NiV-specific therapeutics exist^17^, only supportive care—typically fluids and mechanical ventilation—can be given. While NiV vaccines have shown protection in multiple animal models^18,19^, no candidates have reached the clinical trial stage, and validation will be complicated by the sporadic nature of outbreaks^20^ and a lack of understanding of the correlates of NiV protection^21^. Multiple small molecule drugs, such as Ribavarin and Chloroquine have shown potential in vitro^22,23^, but failed to demonstrate a survival benefit in animal models^24,25^. Recently, monoclonal antibodies (mAbs) have attracted great attention as potential therapeutic agents for viral diseases due to their enhanced potency, extended half-lives, minimal off-targets effects, and most importantly their structure–function relationship^26,27^. Developing a monoclonal antibody against NiV is an ideal solution because administration of the mAb to infected patients and at-risk populations could potentially prevent new infections, treat existing infections, and contain an outbreak.

Indeed, a wealth of structural information on antibody-antigen complexes has been generated in the context of Influenza^28–30^, HIV-1^31,32^, Dengue^33,34^, Zika^35,36^ using X-ray crystallography, which has provided insights into the structural epitope and paratope features. However, significantly less work has been devoted to studying the functional features of epitope and paratope that govern antigen–antibody binding, where the functional residues are those that contribute most significantly to the binding free energy of antibody-antigen interaction. Herein, we investigated the binding interface between m102.4, a potent and cross-reactive mAb against Henipavirus, and NiV glycoprotein (GP)^37^, by first carrying out structure-guided alanine-scanning to map the functional epitope and paratope residues and subsequently generating a model of antigen–antibody interaction using computational docking, which attempts to predict the optimal 3D structure of antigen–antibody interaction starting from their individual structures. Previously, Xu et al.^38^. reported the crystal structure of m102.3 (a derivative of m102.4, with a modified light chain) in complex with HeV GP, which demonstrated that m102.3’s targeted epitope consists of a ring of hydrophilic residues surrounding a central hydrophobic receptor-binding cavity on GP (PDB: 6CMG). Although the NiV GP possesses great sequence similarity to HeV, Xu et al. neither reported epitope-paratope features governing the antibody interaction with NiV GP nor the fingerprint of m102.4 binding with either antigen^38^. The predicted computational model provides a basis for broad recognition and neutralization of various antigens and serves as the starting point for additional antibody reengineering (e.g. affinity maturation) to generate potential therapeutic candidates.

## Results

The membrane anchored GP of NiV is responsible for viral attachment through a protein–protein dominated interaction with the Ephrin-B2/B3 human host cell receptor in which the tip of the G-H loop on the Ephrin fits into a hydrophobic central cavity in the GP^15^. The reported X-ray structure of the m102.3-HeV GP structure indicates that the antibody targets a hydrophilic rim and a hydrophobic central cavity, which is also the binding surface for Ephrin-B2/B3 receptor^38^. Blocking the receptor binding is believed to be the basis of m102.3 (and hence suspected m102.4) viral neutralization (Supplementary Fig. S1).

In order to elucidate the critical hotspots contributing to m102.4′s binding to NiV GP, we performed a structure-guided alanine scanning of epitope and paratope residues. For this study, hotspot residues were defined as those residues (epitope or paratope) for which a ten-fold or greater increase in binding affinity (*K*_d_) was observed (equivalent to a ΔΔG change of 1.36 kcal/mol) relative to WT interaction^39^. Selection of residues for alanine scanning was guided by (1) solvent accessible surface area and (2) knowledge of interfacial residues from m102.3-HeV GP co-crystal structure (PDB: 6CMG). Solvent accessible surface area of antibody and antigen was computed independently using the homology model of m102.4 (Methods) and crystal structure of NiV GP (PDB: 3D11) using DSSP^40^. Residues with > 20% of their areas accessible to the solvent were considered solvent-accessible. The latter filter was applied due to the fact that (1) m102.3 was derived from m102.4, (2) GP of HeV and NiV share > 75% identity and (3) their binding affinity towards HeV (*K*_d_ = 2.74e−8 M and 1.11e−7 M, respectively) and NiV (*K*_d_ = 5.56e−9 M and 2.55e−8 M, respectively) are not vastly dissimilar^38^. Taken together, we hypothesized that m102.4 and NiV GP would employ, at least in part, similar set of residue positions used by m102.3 and HeV, respectively. Starting from the co-crystal structure of m102.3-HeV GP, we defined two residues to be in contact (interfacial residues) if at least two atoms, one from each residue, are within 4.5 Å. Subsequently, we mapped the interfacial residues from PDB: 6CMG onto m102.4 and NiV GP using structure-based sequence alignment^38^. Residues that were solvent accessible and likely to contribute to the binding interface with Nipah GP were considered for alanine scanning. This search process led to a total of 28 and 19 residues on the epitope and paratope surfaces, respectively. We noted that the amino acid sequences of m102.3 and m102.4 published in Xu et al. are different from the published patent^41^. While the reported LC sequences match, there are modifications in the FWR1 and CDR1 regions of the reported HCs (Supplementary Table S4). We employed the m102.4 sequence provided in the patent for recombinant expression and alanine-scanning work. 18/19 residues selected for paratope alanine-scanning are identical between the two published sources. The remaining residue position (31 of VH) has Asn in the sequence published by Xu et al. but Lys in the sequence published by the patent. To probe the involvement of residues, which are alanine in WT epitope or paratope, we mutated alanine to tryptophan as the large aromatic side chain of tryptophan is expected to have the highest disruptive potential to the binding interface^42^. Importantly, 18/19 identified paratope residues are from the heavy chain, which is logical given that the interaction between m102.3 and GP is dominated by the heavy chain.

### Experimental identification of functionally critical residues on GP

We aimed to scan the identified epitope surface to analyze the extent to which various residues were involved in the binding with m102.4. To assay for the functional importance of chosen residues, we tested binding of m102.4 against a panel of recombinantly expressed GP constructs containing mutations to alanine in each of the 28 residues (Methods). We opted to use bio-layer interferometry to determine *K*_d_ values for each GP-mAb combination (Methods). Briefly, after introducing mutations to each construct, we expressed and purified GP mutants with an added C-terminal His tag for purification as well as a signal peptide to aid in secretion (Methods). Next, we tested binding of each of these point mutants to m102.4 (Table 1) and wild-type control.

**Table 1:** Kinetic constants *K*_d_, *k*_a_, and *k*_d_ of the wild-type and mutant GPs.

Mutations	*K*_d_ (nM)	*k*_a_ (M^−1^ s^−1^)	*k*_d_ (s^−1^)
Wild-type	0.07 ± 0.01	6.46 × 10^5^	4.53 × 10^−5^
G238A	0.58 ± 0.23	2.88 × 10^5^	16.6 × 10^−5^
S239A	2.74 ± 0.94	0.30 × 10^5^	8.29 × 10^−5^
C240A	No binding	No binding	No binding
S241A	0.06 ± 0.03	5.50 × 10^5^	2.99 × 10^−5^
R242A	0.15 ± 0.002	2.95 × 10^5^	4.52 × 10^−5^
L305A	0.22 ± 0.08	3.98 × 10^5^	8.17 × 10^−5^
F458A	0.67 ± 0.09	1.77 × 10^5^	11.70 × 10^−5^
P488A	0.14 ± 0.06	3.33 × 10^5^	4.64 × 10^−5^
G489A	1.37 ± 0.14	3.96 × 10^5^	53.5 × 10^−5^
Q490A	0.07 ± 0.005	5.72 × 10^5^	3.92 × 10^−5^
W504A	2.09 ± 0.05	4.16 × 10^5^	87.2 × 10^−5^
E505A	< 0.001	2.00 × 10^5^	< 1.0 × 10^−7^
G506A	No binding	No binding	No binding
V507A	0.02 ± 0.002	3.63 × 10^5^	0.70 × 10^−5^
T531A	< 0.001	3.08 × 10^5^	< 1.0 × 10^−7^
A532W	No binding	No binding	No binding
E533A	< 0.001	1.62 × 10^5^	< 1.0 × 10^−7^
E554A	< 0.001	2.49 × 10^5^	< 1.0 × 10^−7^
D555A	0.09 ± 0.002	3.32 × 10^5^	2.85 × 10^−5^
N557A	No binding	No binding	No binding
A558W	No binding	No binding	No binding
Q559A	< 0.001	6.37 × 10^5^	< 1.0 × 10^−7^
E579A	< 0.001	6.00 × 10^5^	< 1.0 × 10^−7^
Y581A	< 0.001	1.46 × 10^5^	< 1.0 × 10^−7^
T583A	0.06 ± 0.01	5.05 × 10^5^	3.08 × 10^−5^
D585A	0.05 ± 0.01	3.39 × 10^5^	1.85 × 10^−5^
N586A	0.30 ± 0.07	2.50 × 10^5^	7.44 × 10^−5^
I588A	0.36 ± 0.13	1.36 × 10^5^	5.06 × 10^−5^

We observed significantly decreased expression for three mutants (G238, S239, C240). Next, we saw near complete knockouts in binding for five mutants: C240A, G506A, A532A, N557A and A558A (Supplementary Fig. S2). We saw also significant losses in binding (10–40-fold) for S239A, W504A, G489A, F458A (Y458 on HeV, a key difference between the two). These residues comprise a binding sector that fits into the F/Y and L loops of the EFBN2/3 and are part of the known m102.3 epitope on HeV, which also confirms some degree of overlap between the binding sites of m102.3 and m102.4. In total, 9/28 residues were determined to be critical epitope hotspots (Table 1).

### Experimental determination of m102.4 binding features

**Figure 1:**
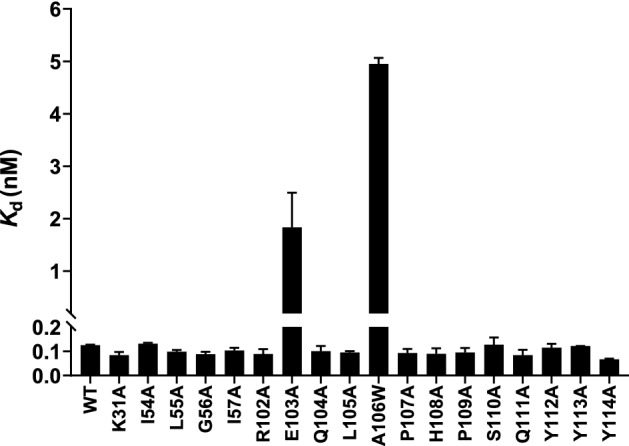
Binding characteristics of mutant Nipah antibodies. Binding of Nipah antibody heavy-chain mutants to the wild-type GP was determined using indirect ELISA. All *K*_d_ values represented the average of three independent experiments and report in nanomolar (nM). Graph was generated using GraphPad Prism version 8.4.3 for Windows.

Next, we carried out site-directed mutagenesis (see Methods) on each of 19 residues (18 on VH and 1 on VL) that are solvent accessible and were previously suggested by the m102.3-HeV GP crystal structure to be involved in the structural epitope. Despite the heavy involvement of heavy chain in GP interface, most mutations made to the heavy chain had little to no impact on NiV GP binding, as measured by indirect ELISA (Methods). Amongst those tested, only two heavy-chain mutants located on HCDR3 showed > tenfold reductions on *K*_d_’ values: E103A and A106W (Fig. 1 and Table 2). Both of these residues are involved in making contacts with the HeV GP epitope surface, however, there was no direct correlation between binding and density of interfacial contacts. E103 on the heavy chain was seen to make a H-bond with S239 on the HeV GP, and A106 was observed to make a hydrophobic interaction with the hydrophobic GP core.

**Table 2:** Binding characteristics of mutant Nipah antibodies. Binding of each Nipah antibody to the wild-type GP was determined using indirect ELISA. The data represent mean ± SD for at least two independent determinations.

	Mutation	*K*_d_ (nM)
VH	WT	0.12 ± 0.002
	K31A	0.08 ± 0.01
	I54A	0.13 ± 0.004
	L55A	0.10 ± 0.007
	G56A	0.09 ± 0.01
	I57A	0.10 ± 0.01
	R102A	0.09 ± 0.02
	E103A	1.83 ± 0.66
	Q104A	0.10 ± 0.02
	L105A	0.10 ± 0.006
	A106W	4.95 ± 0.11
	P107A	0.09 ± 0.02
	H108A	0.09 ± 0.02
	P109A	0.10 ± 0.02
	S110A	0.13 ± 0.03
	Q111A	0.08 ± 0.02
	Y112A	0.11 ± 0.02
	Y113A	0.12 ± 0.001
	Y114A	0.07 ± 0.004
VL	R30A	0.13 ± 0.007

Xu infers that the core of the m102.3-HeV GP interface is formed by van der Waals or hydrophobic contacts (two from HCDR2 (L55 and I57) and five from HCDR3 (L105, P107, P109, S110, Y112)) whereas the periphery of the interface is formed by hydrophilic contacts from H-CDR1 (K31), HCDR2 (G56), HCDR3 (R102, E103, Q111, Y112, Y113, Y114) and L-CDR1 (R30)^38^. Remarkably, apart from E103, we saw no effect on binding upon mutating the above residues in m102.4 (Table 2). It is noteworthy that, A106, which showed the highest impact on binding, was not mentioned by Xu et al.^38^.

We find it significant that the binding interaction between m102.4 and NiV GP is affected only by two paratope hotspot residues. To determine the prevalence of such asymmetric distribution of hotspots, we compiled alanine-scan data from prior studies that explored antibody-antigen interfaces via alanine scanning. For each antibody, the distribution of hotspots on the paratope (H1, H2, H3, L1, L2, L3) was gathered and tabulated as shown in Table 3. For each given antigen, the number of functional epitope residues was gathered from epitope mapping studies, using studies sourced from the Immune Epitope Database (https://www.iedb.org/)^43^. Remarkably, m102.4 is the only example of an antibody with so few hotspots, all locally distributed on a single CDR loop (Table 3). While it is possible that this is a unique feature of antibodies that possess long HCDR3 loop (20 amino acids or longer) and use loop insertion to achieve high affinity binding (e.g. C05, CH65), the lack of alanine scanning data for such antibodies prevent us from making any general conclusions.

**Table 3:** Distribution of hotspot paratope residues from prior alanine-scanning antibody studies.

Ab/Ag	Method of hotspot determination	Number of paratope positions analyzed	Crystal structure	HCDR1	HCDR2	HCDR3	VH	LCDR1	LCDR2	LCDR3	VL
m102.4/NiV GP	Ala-scan + ELISA	28	N/A	0	0	2	2	0	0	0	0
Y0317/VEGF	Phage display + competitive ELISA	68	1CZ8	5	3	6	14	0	0	2	2
HyHEL-10/HEL	Ala-scan + ELISA	12	2DQJ	1	3	1	5	2	1	1	4
b12/HIV gp120	Ala-scan + ELISA	18	1HZH	N/A	N/A	7	7	N/A	N/A	N/A	0
2F5/HIV gp41	Ala-scan + ELISA	22	2F5B	N/A	N/A	4	4	N/A	N/A	N/A	0
Fab2C4/ErbB2	Phage display + ELISA	61	1L7I	4	8	7	19	0	1	1	2
6B4/GP lba	Ala-scan + ELISA	12	N/A	N/A	0	1	1	4	N/A	1	5
N2/alpha-synuclein	Ala-scan + ELISA	18	2X6M	N/A	4	2	6	N/A	N/A	N/A	N/A
Fab 13B8.2/CD4	Spot peptide Ala-scan	16	TBD	1	1	0	2	0	0	0	0
HA22-LR/CD22	Ala-scan + WST-8 assay	36	N/A	0	N/A	4	4	1	0	0	1
HzKR127/HepB	Ala-scan + ELISA	50	2EH8	3	3	2	8	7	1	6	14
82D6A3/VWF	Ala-scan + ELISA	13	2ADF	0	0	2	2	0	0	0	0
Anti-PAI	Ala-scan + SPR (not only single mutants)	26	N/A	1	0	3	4	0	0	5	5
Fab37/HER2	Phage display, saturation mutagenesis	20	3N85	3	1	4	8	N/A	N/A	4	4
bH1/VEGF	Ala-scan + SPR	35	3BE1	0	0	2	2	2	0	1	3
Cetuximab/EGFR	Ala-scan + SPR	27	1YY9	0	1	2	3	0	0	1	1
D1.3/HEL	Ala-scan + SPR	10	1DVF	0	0	2	2	0	0	1	1

Criteria for hotspot determination were for a nearly tenfold impact of mutation on *K*_d_ (equivalent to a 1.36 kcal/mol ddG difference). “N/A” refers to CDR loops that were not scanned in the analysis.

The disparity between the observed structural interface between m102.3-GP (HeV) and experimentally measured hotspots of m102.4 (Supplementary Table S3) prompted us to investigate the degree to which the binding mode of m102.4 is similar to that of m102.3 as observed in the co-crystal structure. Moreover, Xu et al.^38^ showed that many residues outside the contact surface region of m102.3 affect m102.4 binding, raising the possibility that m102.4 binds GP in a slightly different manner compared with m102.3^38^. Furthermore, the LC of m102.4 carries a different set of amino acids in the HC/LC interface than m102.3, which could potentially affect the HC:LC packing, which in turn could alter the paratope structure required for optimal interaction with the antigen (Supplementary Fig. S3).

### Computational protein–protein docking to predict m102.4-GP (NiV) structure

**Figure 2:**
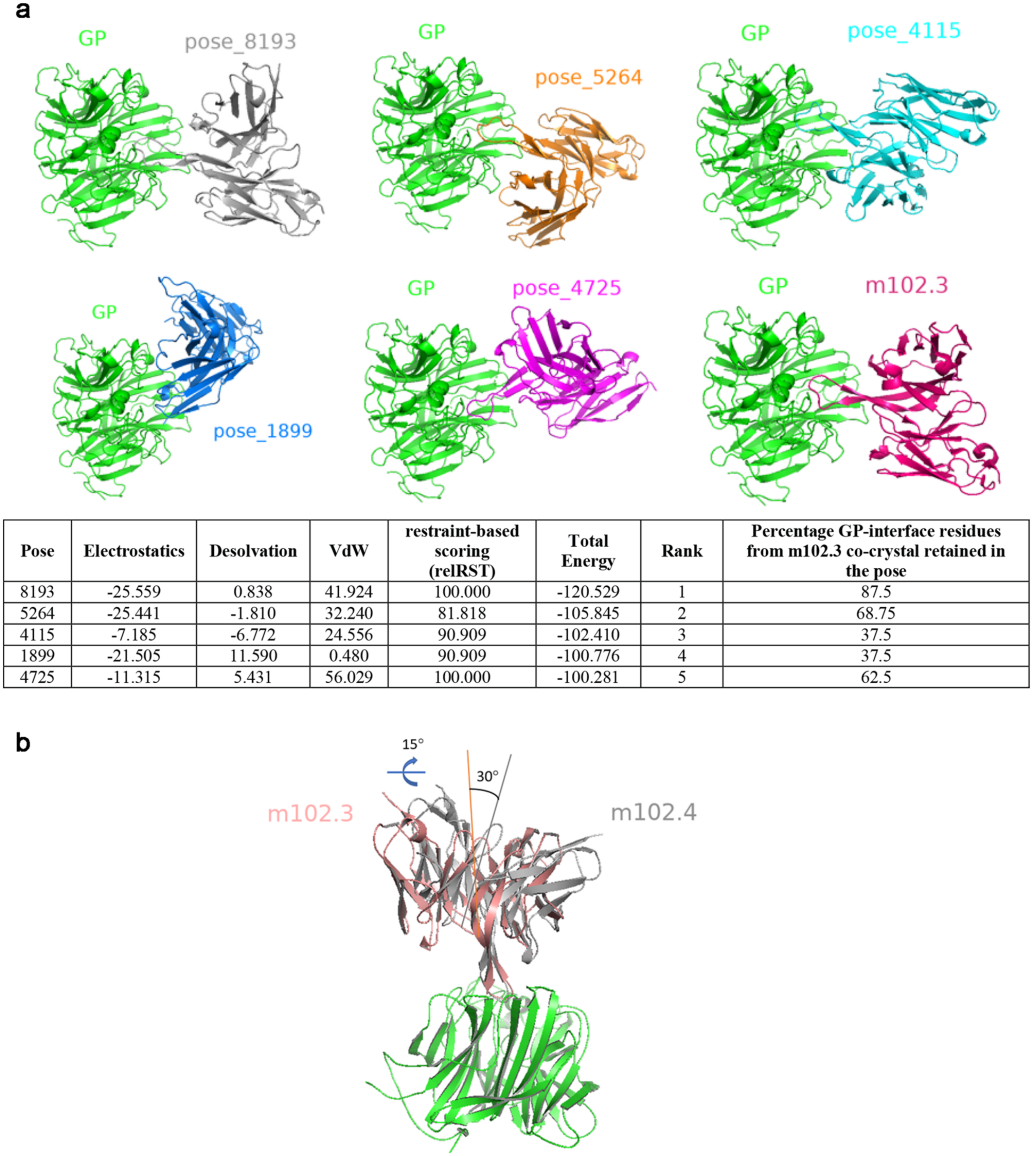
Computational docking and mode of m102.4-GP binding. (**a**) The top five docked models identified by pyDockWeb along with co-crystal of m102.3 (PDB: 6CMG) are shown. The orientation of GP (green) is intact in all six panels. The table underneath shows the energetic terms along with percentage of GP-interface residues from m102.3 co-crystal that are retained in each of the five poses. (**b**) Structural superposition of m102.3 co-crystal (PDB: 6CMG) and pose_8193 using GP (green) as the reference. The angles that capture the differences in binding orientation are highlighted to show the differences. All figures were generated using PyMOL (https://www.pymol.org).

In order to determine a model of m102.4 interaction with NiV GP, we performed antigen–antibody docking with pyDockWEB^44^ starting from the homology model of m102.4 and crystal structure of NiV GP (PDB: 3D11) using constraints from the panel of antigen and antibody mutations that showed significantly lower binding: G238, S239, C240, F458, G489, W504, G506, A532, N557, A558, I588 (epitope) and VH:E103 and VH:A106 (paratope). pyDockWEB has been validated on standard protein–protein docking benchmark 4.0 datasets and is comparable to other docking solutions evaluated by Critical Assessment of PRedicted Interactions (CAPRI) studies^44^. In order to minimally restrain the docking procedure, we did not specify residues to block from being part of the interface. PyDockWEB performs rigid body docking and ranks the poses using electrostatics, desolvation, and van der Waals scoring terms. The docking procedure generated 500 poses in total. The five top ranked poses had m102.4 bound to GP in different orientations (Fig. 2a); each of the top five poses had an overall energy score less than − 100, and the best pose (‘pose_8193’) had a score of –120.529. The orientation of m102.4 in pose_8193 indicated that the mAb was capable of stereo-specifically blocking Ephrin binding, burying all the hotspot residues that were used as constraints (Supplementary Fig. S4). Furthermore, the epitope inferred from pose_8193 was conserved (90.625%) in HeV, supporting the observed antibody cross-reactivity. Other poses did not exhibit both of these properties, enhancing our confidence in the pose_8193 model. Importantly, pose_8193 superimposed with mAb-HeV co-crystal structure (PDB: 6CMG) with an All Atom RMSD of 3.809 Å, suggesting that the mode of engagement of m102.4 is not vastly different from that of m102.3, consistent with the findings of Xu et al.^38^. By contrast, other poses were strikingly different from PDB: 6CMG (Fig. 2a). However, structure superposition of pose_8193 on GP of m102.3 complex (PDB: 6CMG) indicated important nuances in the mode of engagement of the two antibodies. First, the pseudo-twofold axis of the Fv fragment of m102.3 is tilted roughly 30 degrees from the pseudo-twofold axis of m102.4 Fv. Second, when viewed along a line perpendicular to the central axis of the six-blade β-propeller, m102.4 Fv is oriented 15° clockwise relative to m102.3 Fv (Fig. 2b). Despite the above differences, the HCDR3 loop of m102.4 and m102.3 seem to make several overlapping contacts against the GP of NiV (Supplementary Fig. S5). Of note, the footprint of m102.4 on NiV GP is slightly smaller than the footprint of m102.3 on HeV GP (880 Å^2^ vs. 940 Å^2^). Although the HC of both antibodies contribute to similar buried surface area (848.6 Å^2^ for m102.3 vs. 855.3 Å^2^ for m102.4), the differences in orientation affect the contribution of LC (91.3 Å^2^ for m102.3 vs. 24.9 Å^2^ for m102.4). Additionally, 13 H-bonds and 2 salt bridge contacts are seen in the interface of m102.3, with LC contributing to 1 H-bond and both the salt bridge contacts (Supplementary Fig. S6). The latter is mediated by LC:R30. By contrast, only 8-bonds are observed in the m102.4 interface with negligible contributions from the LC. Despite LC:R30 being involved in the m102.4-NiV GP interface, it does not form a salt bridge interaction. This explains why reversal of LC:R30 to alanine in m102.4 does not affect the binding. The increased interface complementarity of m102.3 also correlates with m102.3’s tighter binding to both NiV and HeV^38^.

It must be noted that selecting residues to block from and force into the binding interface enables the search algorithm to narrow on meaningful protein–protein interfaces. The fact that pyDockWEB was able to predict a pose similar to the experimentally characterized structure (PDB: 6CMG) as the top model despite not providing residues to block from interface highlights the promising physiochemical and geometric complementarity of the interface and the power of alanine-scanning data in driving structure prediction.

The interface area buried on the antigen by the antibody generally correlates with the binding affinity. Although recognition of the GP cavity is mediated predominantly by insertion of HCDR3 alongside few contacts in the non-cavity region from HCDR2 and LCDR1, m102.4 binds a relatively large surface region (880 Å^2^) when compared with other antibodies that only use HCDR3 to bind antigen (CH65, C05). This complementarity between the epitope and paratope and the relatively larger interface area helps achieve nanomolar binding and exceptional neutralization potential. Our assessment of publicly available antibody sequences (over 1 million) indicate that less than 2.5% of antibodies possess HCDR3 loops longer than 20 amino acids. Solving the structure of such antibodies bound to their antigen will provide valuable information about the crucial role of HCDR3s in antigen binding.

To determine the breadth of neutralization, we examined epitope sequence diversity contained within all full-length, non-redundant GP sequences in the NCBI GenBank database^45^. Our analysis revealed that the entire structural epitope was 100% conserved in the 13 NiV and 35 HeV strains found in GenBank. It is interesting to note that alanine mutations at several sites on GP (E505, T531, E533, E554, Q559, E579, Y581,) improved the antibody binding affinity by at least an order of magnitude, suggesting that the mAb binding would be enhanced should these alanine mutants arise naturally during the course of viral evolution. Indeed, this also indicates that epitope variation has a significant impact on m102.4’s activity. Upon re-inspection of the m102.4-NiV GP model, many of these sites are located proximal to the hydrophobic or aromatic residues of HCDR3 (L100A, P100C, P100E, Y100H, Y100I), which provides a rationale for the increase in affinity seen upon alanine mutation of the aforementioned epitope sites.

### Mutability of m102.4 epitope and risk of antibody escape

Unlike other viruses such as influenza and HIV, evolutionary pressures have not resulted in significant sequence diversity within members of *Henipavirus* GP family as a result of the short-lived nature of outbreaks and the large time frames between them^1^. This serves as an indication that an evolutionary history is insufficient for identification of residues with high propensities for acquiring mutation on the GP surface. To complement the sequence-based prediction, we computed the significant interaction network (SIN) scores for all the residues on the GP surface (PDB: 3D11) and predicted residues which are constrained to mutate as a result of their “interconnectivity” with other residues^46^ (Methods). As expected, highly constrained residues (with high connectivity with other residues) are seen to be located at buried areas of the protein (Supplementary Fig. S7), consistent with the pattern observed in our previous study^46^. The mean SIN score of m102.4 epitope is slightly lower than the mean score of GP (0.3323 vs. 0.3872). Amongst the poorly networked residues (defined as those having network score < 0.5), alanine-scanning analysis indicates that mutations at G238, S239, G489, G506, A532, N557, A558 lead to detrimental effect on mAb binding, indicating that these are potential sites for emergence of escape mutants. Consistent with the observed nature of the epitope, Xu et al.^38^ reported (through serial passaging in culture) two escape mutants that developed resistance against m102.4 in the NiV and HeV with mutations in residues V507 (V507I) and D582 (D582N), respectively. In alignment with this, both residues possessed relatively low SIN scores, which is a compelling indication that SIN analysis accurately captures the “evolvability” of residues in this viral system. We did not sample D582 because it did not seem to make any direct contact in the co-crystal structure. Contrary to Xu et al., mutation at V507 did not cause any impact but this is perhaps due to the conservative nature of substitution with an amino acid of similar or reduced size, with no steric issues (unlike V507I). The other three residues that impact binding upon mutating to alanine (F458, W504, I588) have network scores > 0.5, suggesting that there is a significant structural penalty to mutation and thus these residues are unlikely to be the sites of antibody escape mutants.

In summary, the integrated analysis of alanine-scanning and computational approach has allowed us to predict the structural model of m102.4-GP (NiV) complex and explain its neutralization breadth. This overall methodology can be universally applied to study several antibody-antigen interactions for which X-ray crystal structures do not exist.

## Discussion

mAbs play a vital role in biopharmaceuticals owing to their high antigen affinity, specificity and long half-life^47^. While rational engineering of mAbs to improve their binding affinities and specificities is highly desirable, this effort is also typically dependent on the availability of high-resolution antigen–antibody X-ray structures. Compared to ab initio protein–protein docking, computational docking guided by experimentally available data about the interface (e.g. alanine-scanning mutants, NMR epitope mapping) have shown considerable promise in accurately predicting antigen–antibody structure^48^.

Application of structure-guided alanine scanning to m102.4-GP (NiV) interface indicated that the interaction is dominated by just two HCDR3 residues, which is unusually low for an antigen–antibody interface. Selection of paratope residues for alanine-scanning was driven by solvent accessible surface area and knowledge of m102.3’s binding interface with HeV GP. Although we cannot rule out the possibility that our selection criteria excluded paratope residues that contribute to the interface through indirect effects, we believe such residues are less likely to have a significant impact on binding affinity (greater than ten-fold). Furthermore, the computationally docked model of m102.4 indicated differences in the positioning of Fv relative to m102.3, although the core interfacial contacts were preserved. Xu et al. indicated that many residues outside the contact surface region of m102.3 affect m102.4 binding, raising the possibility that m102.4 binds GP in a different manner compared with m102.3^38^. However, the results of our alanine scanning analysis and computational docking indicate otherwise. We speculate that the distal mutations (Xu et al.^38^) affect m102.4 binding through allosteric effects. While it will be useful to compare the antibody-bound form of GP from pose_8193 with the receptor-bound form of GP from the solved co-crystal structure^49^, however, existing docking methods do not accurately capture binding-induced conformation changes^50^. We therefore did not undertake this effort.

The structure of m102.4-GP predicted herein could serve as a basis for further optimization of m102.4. For instance, given the lack of importance of the m102.4 light chain, heavy-chain only antibody variants (e.g. VHH or nanobodies) could potentially be explored. Since m102.4 interaction with GP seems to be dominated by HCDR3 alone, cyclic peptides based on HCDR3 loop of m102.4 could be explored as potential mimetics. Cyclic peptides based on broadly neutralizing antibodies have been successfully engineered against the highly conserved hydrophobic groove at the HA1/HA2 interface of hemagglutinin^51^.

## Methods

### Significant Interactions network: identification of mutationally constrained regions

We previously developed and validated a method to predict highly “networked” residues in a protein starting from its 3D crystal structure or homology model^46^. Briefly, our method maps the protein as a 2-dimensional network (using available crystal structures) and captures the “interconnectivity” of each amino acid residue. This is quantified by assigning each residue a score describing the extent of its inter-residue atomic interactions. Residues with high levels of interconnectivity are therefore constrained to not mutate, given their structural importance.

Using the previously developed algorithm^46^, we carried out Network Analysis on the NiV GP (PDB: 3D11). Briefly, this method projects proteins as a network from available crystal structure, with the “nodes” as residues and “edges” as interatomic interactions. By carrying out a weighted sum of all the interatomic interactions (both covalent and noncovalent), each residue is given a normalized “Network Score.” This score quantifies the extent of a residue’s interconnectivity; thus, a low Network Score residues indicates that a residue is not very involved in the local structure of the protein and can thus be easily mutated without much loss from a structural standpoint. However, high Network Score residues are highly interconnected, having great structural burden and low potential for mutation.

### Homology modeling of m102.4

The homology model of m102.4 was generated using AbodyBuilder (https://opig.stats.ox.ac.uk/webapps/sabdab-sabpred/Modelling.php). Under default settings, the framework, H1, H2, L1 and L2 were modeled using PDB:4M62 template whereas H3 and L3 were modeled using 6CMG and 2XRA, respectively. The homology modeling was evaluated using the Swiss PDB Viewer (https://spdbv.vital-it.ch/) for stereochemical outliers. The model of m102.4 superposed with the Fv in the co-crystal with an RMSD of 0.649, indicating appreciable structural similarity. No significant differences were observed in the VH:VL orientation. Since 6CMG was used as a template for HCDR3, the loop adopts a similar β-hairpin conformation, as expected.

### Site-directed mutagenesis

To study epitope-paratope interaction, site-directed mutagenesis was performed to obtain twenty-eight GPs, eighteen of antibody heavy-chain, and one antibody light-chain mutants. The primers listed in (Supplementary Tables S1 and S2) were used to introduce an amino acid alanine or tryptophan to the target DNA sequences. PCR was performed in total 30 μL reaction containing 1 × Phusion High-Fidelity PCR Master Mix (Thermo Fisher Scientific), 0.8 μM each forward and reverse primer (Integrated DNA technologies), and specified amount of the target DNA. The PCR cycling conditions were as follows: 95 °C for 3 min; followed by 35 cycles of 95 °C for 10 s, annealing of primers for 30 s at annealing temperature of 60 °C, and 30 s at 72 °C for extension. The PCR products were detected using an agarose gel electrophoresis. After gel elution, the PCR products with overlapping ends were added into a 20 μL Gibson assembly reaction (New England Biolabs) along with linearized pcDNA3.1(−) (Invitrogen). The resulting recombinant plasmids were transformed into the bacterial strain DH5α and sequenced to check for the presence of an amino acid alanine or tryptophan.

### Histidine-tagged GP cloning and purification

The hexa-histidine tag was introduced to the C-terminus of wild-type and twenty-eight GP mutants. The forward primer, gPEcoRI, was designed with *Eco*RI restriction site on 5′ end. Eighteen nucleotides coding six histidine residues were added to the 3′ end of reverse primer, gPHistag. The PCR cycling conditions were as follows: 95 °C for 3 min; followed by 35 cycles of 95 °C for 10 s, annealing of primers for 30 s at annealing temperatures of 60 °C, and 1 min at 72 °C for extension. The PCR product was cloned into pcDNA3.1(+) (Invitrogen) at *Eco*RI and *Eco*RV sites. Positive clones were screened using restriction digestion of the isolated plasmid and further confirmed by sequencing. Expression of His-tagged GP was carried out in HEK 293-F FreeStyle suspension cells (Invitrogen) grown in FreeStyle 293 Expression Medium (Invitrogen) and maintained at 37 °C, 80% humidity and 8% CO_2_. Cells were transiently transfected with the GP plasmids using Polyethyleneimine Max (PEI-MAX, PolySciences) and were harvested seven days after transfection. The supernatant was collected by centrifugation, filtered through Acrodisc Syringe Filters 0.2 µm with Supor Membrane (Pall), and purified using Amintra Ni–NTA Affinity Resin (Expedeon). The beads were washed with 15 mM imidazole in lysis buffer and the bound protein was eluted with 150 mM imidazole in lysis buffer. Fractions were collected, and buffer exchanged to phosphate-buffered saline (PBS) using Amicon Ultra Centrifugation Filters (Merck). Proteins were subsequently analyzed by SDS-PAGE in 0.75 mm-thick SDS-PAGE gels containing 10% acrylamide. Gels were run at 100 V and stained using InstantBlue (Expedeon).

### Antibody expression and purification

Similar to the method for expressing GPs^52^, pcDNA 3.1(−) expression vectors containing encoding gene for different anti-Nipah mAbs was transiently transfected into HEK 293-F FreeStyle cells. After seven days, supernatant was harvested, filtered, and purified using Pierce ProteinA Agarose (Thermo Fisher Scientific). The beads were washed with PBS and the bound protein was eluted with 0.1 M glycine pH 2.0 and neutralized with 1 M Tris pH 8.0. Fractions containing antibody were collected and buffer exchanged into PBS. Proteins were assessed using NuPAGE 4–12% Bis–Tris Protein Gels (Invitrogen) followed by InstantBlue Protein Stain (Expedeon) to confirm purity.

### Enzyme-linked immunosorbent assays (ELISA)

ELISA protocol was adapted from Quinlan et al.^52^. Briefly, 100 μL of the wild-type GP in PBS was adsorbed to clear 96-well Maxisorp plates (Nunc) at a concentration of 1 μg/mL and left at 4 °C overnight. Plates were washed with PBS with 0.05% Tween 20 (PBST) and incubated with 100 μL of 2% BSA in PBST for 1 h at room temperature. Plates were washed with PBST then threefold serially diluted of anti-Nipah mAb were added to wells followed by incubation for 2 h. After washing with PBST, 100 μL of secondary antibody, goat anti-human IgG H&L conjugated with HRP (1:10,000, Abcam) were incubated for 1 h. Finally, plates were washed, and incubated with 100 μL of TMB substrate (KPL) for 5 min and quenched with 100 μL of 1 N H_2_SO_4_. Plates were read using a Tecan Infinite N200 Pro plate reader at 450 nm.

### Binding studies performed on Octet QKe

All kinetic assays on Octet QKe (ForteBio) were performed according to the company’s recommendations^53^ using 96-well plates and samples were diluted in freshly prepared PBS with 0.1% Tween 20, pH 7.4 (PBST). Kinetic assays were performed by first capturing biotinylated wild-type Nipah antibody using streptavidin biosensor (ForteBio) followed by two baseline steps in PBST buffer with orbital shake speed of 1000 rpm. The antibody-captured biosensors were then submerged in wells containing different concentrations of GP for 120 s followed by varied period of dissociation in PBST buffer. The binding sensorgrams were collected using the high sensitivity 8-channel detection mode on the Octet QKe biosensor.

## Supplementary information

Supplementary Information.
