# Supplementary material for: Prediction of the binding interface between monoclonal antibody m102.4 and Nipah attachment glycoprotein using structure-guided alanine scanning and computational docking
Source: Sci Rep. 2020 Oct 26;10:18256. doi: 10.1038/s41598-020-75056-y (PMC7588459; doi:10.1038/s41598-020-75056-y)
Supplement: Supplementary file 1 — Supplementary Information. [file 41598_2020_75056_MOESM1_ESM.pdf]

Prediction of the binding interface between monoclonal antibody m102.4 and Nipah attachment glycoprotein using structure-guided alanine scanning and computational docking

Phanthakarn Tit-oon[1,#], Kannan Tharakaraman[2,3,#], Charlermchai Artpradit[1,#], Abhinav Godavarthi[1,π], Pareenart Sungkeeree[1], Varun Sasisekharan[1], Jarunee Kerdwong[1], Nathaniel Loren Miller[2,3,4], Bhuvna Mahajan[1], Amnart Khongmanee[1], Mathuros Ruchirawat[1], Ram Sasisekharan[2,3,*], and Mayuree Fuangthong[1,*]

[1]Translational Research Unit, Chulabhorn Research Institute, Bangkok, 10210, Thailand
[2]Koch Institute for Integrative Cancer Research, Massachusetts Institute of Technology, Cambridge, MA 02139, USA
[3]Department of Biological Engineering, Massachusetts Institute of Technology, Cambridge, MA 02139, USA
[4]Harvard-MIT Division of Health Sciences & Technology, Massachusetts Institute of Technology, Cambridge, MA 02139, USA
[π]Present address: Yale University, New Haven, CT 06520, USA

[#]These authors contributed equally to this work.

*To whom the correspondence should be addressed.

Email: mayuree@cri.or.th; Tel: +66 2 553 8535; Fax: +66 2 553 8558;

Email: rams@mit.edu; Tel: +1617 258 9494

SUPPLEMENTARY MATERIALS

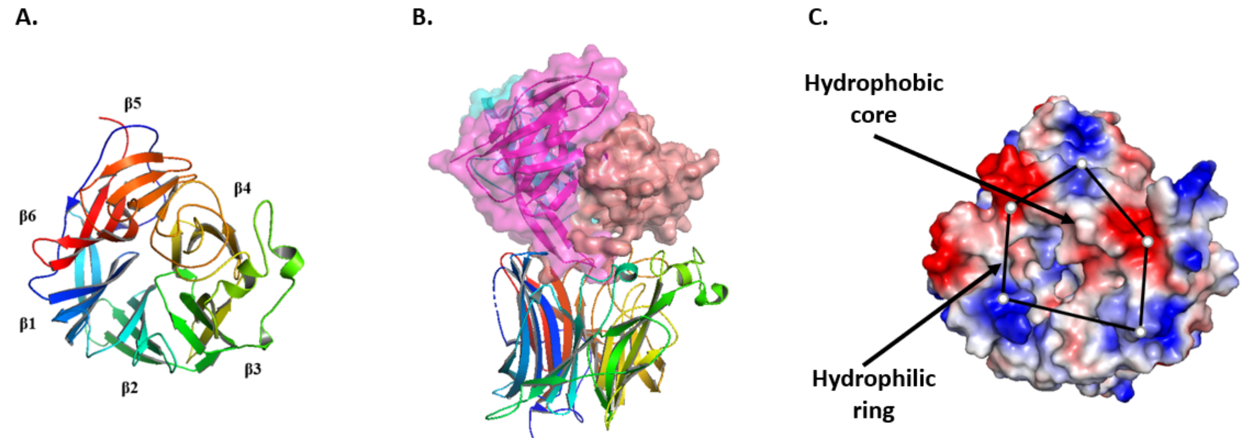

Supplementary Figure S1. Hendra GP structure and m102.3 epitope. (A) the toroidal arrangement of deglycosylated GP formed by the six beta propeller blades around a central axis (labeled). The view of GP is along the line of central axis. GP is colored in rainbow color with the N terminus in blue and the C terminus in red; (B) GP bound to m102.3 (PDB: 6CMG) and its receptor Ephrin-B2 (PDB: 6PDL). View of the GP is perpendicular to the central axis. The Fv of m102.3 is colored in magenta and cyan, respectively, whereas the receptor is in light brown. (C) m102.3 epitope consisting of hydrophobic core in the center and hydrophilic pockets at the periphery highlighted on the electrostatic surface of GP.

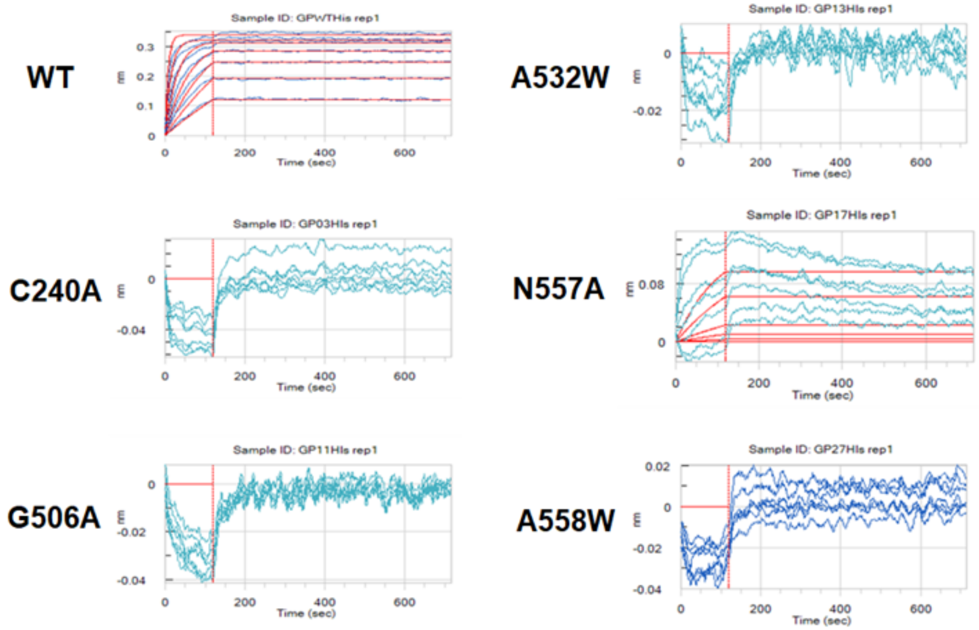

Supplementary Figure S2. Sensorgram data showing fit curves (shown in red) generated from Octet QKe. Kinetic assays were performed by first capturing biotinylated wild-type Nipah antibody using streptavidin biosensor. The antibody-captured biosensors were then submerged in wells containing different concentrations of G-protein for 120 sec followed by dissociation period in PBST buffer. The wild-type G-protein generates signals that fit perfectly to the curves. No fit curves were generated from the mutant G-protein with C240A, G506A, A532W, N557A, or A558W mutations.

```
                                               *
m102.3      EIVMTQSPGTPSLSPGERATLSCRASQSIRSTYLAWYQQKPGQAPRLLIYGASSRATGIP
m102.4      EIVMTQSPGTLSLAPGERATLSCWASQSVRNNYLAWYQQKPGQAPRLVIYNGSTRATGIP

                                               ****   *
m102.3      DRFSGSGSGTDFTLTISRLEPEDFAVYYCQQYGRSP--SFGQGTKVEIK
m102.4      DRFSGSGSGTDFTLTISRLDPEDFAVYYCQQYGNSRRVTFGGGTKVEIK
```

Supplementary Figure S3. Sequence alignment between LC of m102.3 and m102.4. Amino acid differences are indicated in red. Amino acids that are different in the two chains and fall in the VH:VL interface are highlighted by an asterisk symbol. Chothia CDR loops are colored in yellow.

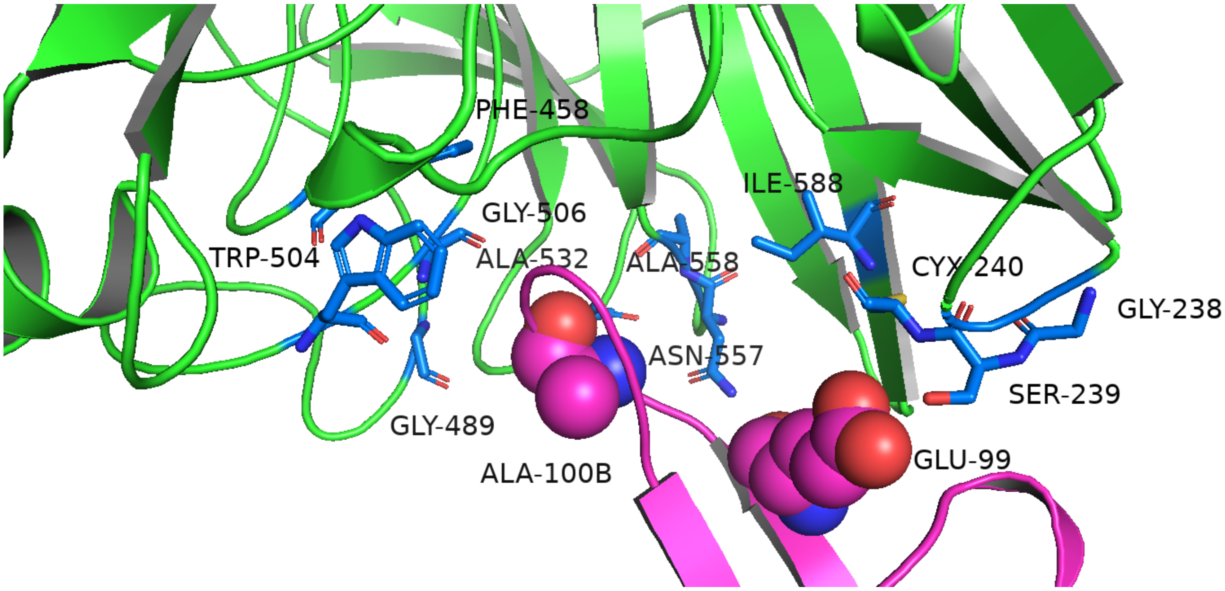

Supplementary Figure S4. Close-up view of m102.4-GP (NiV) interface from the docked model. The residues used as constraints for docking are highlighted by spheres (paratope) and sticks (epitope). The light chain is not seen in this orientation. The heavy chain of m102.4 and GP are colored in magenta and green, respectively.

EGVSNLVGLPNNICLQKTSNQILKPKLISYTLPVVGQSGTCITDPLLAMDEGYFAYSHLE

RIGSCSRGVSKQRIIGVGEVLDRGDEVPSLFMTNVWTPPNPNTVYHCSAVYNNEFYYVLC

AVSTVGDPILNSTYWSGSLMMTRLAVKPKSNGGGYNQHQLALRSIEKGRYDKVMPYGPSG

IKQGDTLYFPAVGFLVRTEFKYNDSNCPITKCQYSKPENCRLSMGIRPNSHYILRSGLLK

YNLSDGENPKVVFIEISDQRLSIGSPSKIYDSLGQPVFYQASFSWDTMIKFGDVLTVNPL

VVNWRNNTVISRPGQSQCPRFNTCPEICWEGVYNDAFLIDRINWISAGVFLDSNQTAENP

VFTVFKDNEILYRAQLASEDTNAQKTITNCFLLKNKIWCISLVEIYDTGDNVIRPKLFAV

KIPEQCTA

Supplementary Figure S5. m102.3 and m102.4 epitope residues mapped on NiV GP sequence. Epitope residues that are common to both antibodies are colored in purple, whereas amino acids unique to m102.3 and m102.4 are in red and blue, respectively. Residues involved in H-bonds or salt bridge contact with both antibodies are indicated by downward pointing black arrows; Residues involved in H-bonds or salt bridge contact with m102.3 or m102.4 but not both are indicated by yellow (m102.3) and green (m102.4) arrows, respectively.

A.

```
NiV-GP       EGVSNLVGLPNNICLQKTSNQILKPKLISYTLPVVGQSGTCITDPLLAMDEGYFAYSHLE
HeV-GP       QGVSDLVGLPNQICLQKTTSTILKPRLISYTLPINTREGVCITDPLLAVDNGFFAYSHLE

NiV-GP       RIGSCSRGVSKQRIIGVGEVLDRGDEVPSLFMTNVWTPPNPNTVYHCSAVYNNEFYYVLC
HeV-GP       KIGSCTRGIAKQRIIGVGEVLDRGDKVPSMFMTNVWTPPNPSTIHHCSSTYHEDFYYTLC

NiV-GP       AVSTVGDPILNSTYWSGSLMMTRLAVKPKSNGGGYNQHQLALRSIEKGRYDKVMPYGPSG
HeV-GP       AVSHVGDPILNSTSWTESLSLIRLAVRPKSDSGDYNQKYIAITKVERGKYDKVMPYGPSG

NiV-GP       IKQGDTLYFPAVGFLVRTEFKYNDSNCPITKCQYSKPENCRLSMGIRPNSHYILRSGLLK
HeV-GP       IKQGDTLYFPAVGFLPRTEFQYNDSNCPIIHCKYSKAENCRLSMGVNSKSHYILRSGLLK

NiV-GP       YNLSDGENPKVVFIEISDQRLSIGSPSKIYDSLGQPVFYQASFSWDTMIKFGDVLTVNPL
HeV-GP       YNLSLGGDIILQFIEIADNRLTIGSPSKIYNSLGQPVFYQASYSWDTMIKLGDVDTVDPL

NiV-GP       VVNWRNNTVISRPGQSQCPRFNTCPEICWEGVYNDAFLIDRINWISAGVFLDSNQTAENP
HeV-GP       RVQWRNNSVISRPGQSQCPRFNVCPEVCWEGTYNDAFLIDRLNWVSAGVYLNSNQTAENP

NiV-GP       VFTVFKDNEILYRAQLASEDTNAQKTITNCFLLKNKIWCISLVEIYDTGDNVIRPKLFAV
HeV-GP       VFAVFKDNEILYQVPLAEDDTNAQKTITDCFLLENVIWCISLVEIYDTGDSVIRPKLFAV

NiV-GP       KIPEQCTA
HeV-GP       KIPAQCSE
```

B.

```
m102.4-VH EVQVIQSGADVKKPGSSVKVSCKSSGGTFSKYAINWVRQAPGQGLEWMGGIIPILGIANY
m102.3-VH EVQLVQSGAEVKKRGSSVKVSCKSSGGTFSNYAINWVRQAPGQGLEWMGGIIPILGIANY

m102.4-VH AQKFQGRVTITTDESTSTAYMELSSLRSEDTAVYYCARGWGREQLAPHPSQYYYYYYGMD
m102.3-VH AQKFQGRVTITTDESTSTAYMELSSLRSEDTAVYYCARGWGREQLAPHPSQYYYYYYGMD

m102.4-VH VWGQGTTVTVSS
m102.3-VH VWGQGTTVTVSS


m102.4-VL EIVMTQSPGTLSLAPGERATLSCWASQSVRNNYLAWYQQKPGQAPRLVIYNGSTRATGIP
m102.3-VL EIVMTQSPGTPSLSPGERATLSCRASQSIRSTYLAWYQQKPGQAPRLLIYGASSRATGIP

m102.4-VL DRFSGSGSGTDFTLTISRLDPEDFAVYYCQQYGNSRRVTFGGGTKVEIK
m102.3-VL DRFSGSGSGTDFTLTISRLEPEDFAVYYCQQYG--RSPSFGQGTKVEIK
```

Supplementary Figure S6. Residues involved in antibody–antigen interactions: m102.3-HeV (PDB:6CMG) and m102.4-NiV (pose_8193). **A**. Antibody contacts on the amino acid sequence alignment of NiV and HeV GP. Contacts made by VH and VL are colored in red and blue, respectively. **B**. Antigen contacts on the amino acid sequence alignment of VH and VL of m102.4 and m102.3, respectively. VH and VL-based contacts are colored in red and blue, respectively, consistent with the coloring employed in **A**.

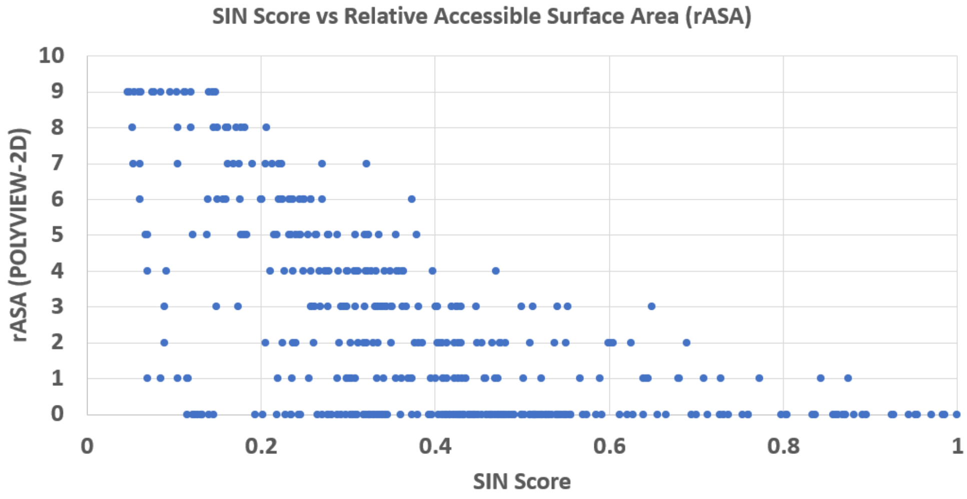

Supplementary Figure S7. Scatter plot of SIN scores and solvent accessibility (expressed as relative accessible surface area calculated from POLYVIEW-2D, http://polyview.cchmc.org/) computed for the X-ray crystal structure of Nipah GP (PDB: 3D11).

Supplementary Table S1. Sequence of DNA primers for G-protein mutation using Gibson assembly.

Purpose	Primer ID - Sequence (5'-3')
G238A mutation	gP007-CGAAAGAATCGCTTCGTGCTCGC
	gP008-GCGAGCACGAAGCGATTCTTTCG
S239A mutation	gP009-AAGAATCGGCGCATGCTCGCGGG
	gP010-CCCGCGAGCATGCGCCGATTCTT
C240A mutation	gP011-AATCGGCTCGGCTTCGCGGGGGG
	gP012-CCCCCCGCGAAGCCGAGCCGATT
S241A mutation	gP013-CGGCTCGTGCGCACGGGGGGTGT
	gP014-ACACCCCCCGTGCGCACGAGCCG
R242A mutation	gP015-CTCGTGCTCGGCAGGGGTGTCAAAG
	gP016-CTTTGACACCCCTGCCGAGCACGAG
L305A mutation	gP017-AGATCCTATTGCAAACTCCACCTACTGGTCCGGTTC
	gP018-GAACCGGACCAGTAGGTGGAGTTTGCAATAGGATCT
F458A mutation	gP019-CCAAGCGTCCGCTTCCTGGGACAC
	gP020-GTGTCCCAGGAAGCGGACGCTTGG
P488A mutation	gP053-GGCACTGGCTCTGTCCGGCCCGGGAG
	gP054-CTCCCGGGCCGGACAGAGCCAGTGCC
G489A mutation	gP055-GGCACTGGCTCTGTGCGGGCCGGGAG
	gP056-CTCCCGGCCCGCACAGAGCCAGTGCC
Q490A mutation	gP021-CCGGCCCGGAGCCAGCCAGTGCC
	gP022-GGCACTGGCTGGCTCCGGGCCGG
W504A mutation	gP023-GGAAATCTGCGCCGAGGGGGTGTACAATG
	gP024-CATTGTACACCCCCTCGGCGCAGATTTCC
E505A mutation	gP025-AATCTGCTGGGCAGGGGTGTACAATG
	gP026-CATTGTACACCCCTGCCCAGCAGATT
G506A mutation	gP027-CTGCTGGGAGGCTGTGTACAATG
	gP028-CATTGTACACAGCCTCCCAGCAG
V507A mutation	gP057-GGCGTCATTGTATGCCCCCTCCCAG
	gP058-CTGGGAGGGGGCATACAATGACGCC
T531A mutation	gP029-TAGCAACCAGGCCGCGGAGAACC
	gP030-GGTTCTCCGCGGCCTGGTTGCTA
A532W mutation	gP031-CAACCAGACCTGGGAGAACCCAG

	gP032-CTGGGTTCTCCCAGGTCTGGTTG
E533A mutation	gP033-CCAGACCGCGGCTAACCCAGTGT<br>gP034-ACACTGGGTTAGCCGCGGTCTGG
E554A mutation	gP035-ACTGGCGTCGGCTGACACCAACG<br>gP036-CGTTGGTGTCAGCCGACGCCAGT
D555A mutation	gP037-GGCGTCGGAGGCCACCAACGCTC<br>gP038-GAGCGTTGGTGGCCTCCGACGCC
N557A mutation	gP039-GGAGGACACCGCCGCTCAAAAGACCATTACCAACTGTTTTC<br>gP040-GAAAACAGTTGGTAATGGTCTTTTGAGCGGCGGTGTCCTCC
A558W mutation	gP059-CAGTTGGTAATGGTCTTTTGCCAGTTGGTGTC<br>gP060-GACACCAACTGGCAAAAGACCATTACCAACTG
Q559A mutation	gP041-CACCAACGCTGCCAAGACCATTACCAACTG<br>gP042-CAGTTGGTAATGGTCTTGGCAGCGTTGGTG
E579A mutation	gP061-GGTGTCGTAGATTGCCACGAGGGAG<br>gP062-CTCCCTCGTGGCAATCTACGACACC
Y581A mutation	gP043-CGTGGAAATCGCCGACACCGGTG<br>gP044-CACCGGTGTCGGCGATTTCCACG
T583A mutation	gP045-AATCTACGACGCAGGTGATAACG<br>gP046-CGTTATCACCTGCGTCGTAGATT
D585A mutation	gP047-CGACACCGGTGCTAACGTCATTC<br>gP048-GAATGACGTTAGCACCGGTGTCG
N586A mutation	gP049-CACCGGTGATGCAGTCATTCGCC<br>gP050-GGCGAATGACTGCATCACCGGTG
I588A mutation	gP051-TGATAACGTCGCCCGCCCTAAACTG<br>gP052-CAGTTTAGGGCGGGCGACGTTATCA
Forward primer for Gibson cloning	fw-CGGCCGCCACTGTGCTGGATTCTAGAGGATCGAACCCTTCAC
Reverse primer for Gibson cloning	gPRv-TGTGGTGGAATTCTGCAGATGAATTCATCATTTCCCCGGGGAC
Forward primer for His-tag cloning	gPEcoRI-GGTGGAATTCTCTAGAGGATCGAACCCTTCAC
Reverse primer for His-tag cloning	gPHistag-TCATCAATGATGATGATGATGATGTGTGCACTGCTCGGGGATC

Supplementary Table S2. Sequence of DNA primers for Nipah antibody mutation using Gibson assembly.

Purpose	Primer ID - Sequence (5'-3')
K31A mutation	Nmab007-AACCTTCTCCGCCTACGCGATTAACTGGGTCCGC
	Nmab008-GCGGACCCAGTTAATCGCGTAGGCGGAGAAGGTT
L55A mutation	Nmab009-CATCCCAATTGCAGGGATCGCCAACTACGC
	Nmab010-CTGTCCCCAGACATCCATCCCGTAGTAATAGGCGTAGTATTGG
G56A mutation	Nmab011-CCCAATTCTGGCAATCGCCAACTACG
	Nmab012-CGTAGTTGGCGATTGCCAGAATTGGG
I57A mutation	Nmab013-AATTCTGGGGGCCGCCAACTACGC
	Nmab014-GCGTAGTTGGCGGCCCCCAGAATT
R102A mutation	Nmab015-GGGTTGGGGAGCTGAACAGTTGGCG
	Nmab016-CGCCAACTGTTCAGCTCCCCAACCC
E103A mutation	Nmab017-TTGGGGAAGGGCCCAGTTGGCGC
	Nmab018-GCGCCAACTGGGCCCTTCCCCAA
Q104A mutation	Nmab019-GGGAAGGGAAGCCTTGGCGCCCCACCCG
	Nmab020-CGGGTGGGGCGCCAAGGCTTCCCTTCC
L105A mutation	Nmab021-AAGGGAACAGGCTGCGCCCCACCCGTC
	Nmab022-GACGGGTGGGGCGCAGCCTGTTCCCTT
A106W mutation	Nmab023-GGAACAGTTGTGGCCCCACCCGTCCCAATAC
	Nmab024-GTATTGGGACGGGTGGGGCCACAACTGTTCC
P107A mutation	Nmab025-ACAGTTGGCGGCCCACCCGTCCC
	Nmab026-GGGACGGGTGGGCCGCCAACTGT
H108A mutation	Nmab027-GTTGGCGCCCGCTCCGTCCCAATAC
	Nmab028-TATTGGGACGGGGCGGGCGCCAAC
P109A mutation	Nmab029-GGCGCCCCACGCTTCCCAATACT
	Nmab030-AGTATTGGGAAGCGTGGGGCGCC
S110A mutation	Nmab031-GCCCCACCCGGCACAATACTACT
	Nmab032-AGTAGTATTGTGCCGGGTGGGGC
Q111A mutation	Nmab033-CCACCCGTCCGCATACTACTACTATTACTAC
	Nmab034-GTAGTAATAGTAGTAGTATGCGGACGGGTGG
Y112A mutation	Nmab035-CCCGTCCCAAGCTTACTACTATTACTACGGGATGGATG
	Nmab036-CATCCATCCCGTAGTAATAGTAGTAAGCTTGGGACGGG

Purpose	Primer ID - Sequence (5’-3’)
Y113A mutation	Nmab037-GTCCCAATACGCCTACTATTACTACGGGATGGATGTCTGGG<br>Nmab046-CCCAGACATCCATCCCGTCGTAATAGTAGGCGTATTGGGAC
Y114A mutation	Nmab039-CCAATACTACGCCTATTACTACGGGATGGATGTCTGGGGACAG<br>Nmab040-CTGTCCCCAGACATCCATCCCGTAGTAATAGGCGTAGTATTGG
L55A mutation	Nmab048-CATCATCCCAGCACTGGGGATCGCC<br>Nmab047-GGCGATCCCCAGTGCTGGGATGATG
Forward primer for Gibson assembly	fw-CGGCCGCCACTGTGCTGGATTCTAGAGGATCGAACCCTTCAC
Reverse primer for Gibson assembly	VHrv-<br>TGTGGTGGAATTCTGCAGATTCATCATTTACCCGGCGACAACGACAGTG

Supplementary Table S3. Theoretical prediction of changes in Gibbs free energy of binding ($\Delta\Delta G$) computed using mCSM-AB[1]. The positions inferred by Xu *et al.*[2] are highlighted in yellow. As seen below, 7/13 alanine mutations reduce antigen binding, contradicting the experimentally generated data.

CHAIN	WILD_RES	RES_POS	MUT_RES	RSA	PRED_ ΔΔG	Predicted Affinity Change
B	R	30	A	58.0	-0.146	Reduce
B	T	32	A	66.6	-0.658	Reduce
B	R	94	A	58.7	-1.572	Reduce
C	N	31	A	64.5	0.152	Increase
C	I	54	A	48.9	-0.336	Reduce
C	G	56	A	88.0	0.191	Increase
C	I	57	A	11.0	-0.291	Reduce
C	R	102	A	47.0	-1.662	Reduce
C	E	103	A	8.2	-1.503	Reduce
C	Q	104	A	9.9	-0.575	Reduce
C	L	105	A	0.6	0.643	Increase
C	A	106	W	0.0	-1.257	Reduce
C	P	107	A	0.1	-0.297	Reduce
C	H	108	A	9.7	0.825	Increase
C	P	109	A	3.3	0.26	Increase
C	S	110	A	32.4	0.079	Increase
C	Q	111	A	58.3	0.035	Increase
C	Y	112	A	11.4	-0.304	Reduce
C	Y	113	A	9.4	-0.951	Reduce
C	Y	114	A	8.8	-1.242	Reduce
C	Y	115	A	16.5	-1.518	Reduce

**RSA: Relative Solvent Accessibility

Supplementary Table S4. Amino acid sequences of VH and VL regions of m102.3 and m102.4 disclosed in US 2009/0214428 A1 (2009) and Xu *et al.*[2]. Amino acid differences between the two published sources are highlighted in bold and colored in red.

Mab	Source	VH	VL
m102.3	US 2009/0214428 A1 (2009)	EVQ**VI**QSGA**D**VKK**P**GSSVKVSCKSSGGTFS**K**<br>YAINWVRQAPGQGLEWMGGIIPILGIANYAQ<br>KFQGRVTITTDESTSTAYMELSSLRSEDTAV<br>YYCARGWGREQLAPHPSQYYYYYYGMDVWGQ<br>GTTVTVSS	EIVMTQSPGTPSLSPGERATLSCRASQ<br>SIRSTYLAWYQQKPGQAPRLLIYGASS<br>RATGIPDRFSGSGSGTDFTLTISRLEP<br>EDFAVYYCQQYGRSPSFGQGTKVEIK
	Xu *et al.*[2]	EVQ**LV**QSGA**E**VKK**R**GSSVKVSCKSSGGTFS**N**<br>YAINWVRQAPGQGLEWMGGIIPILGIANYAQ<br>KFQGRVTITTDESTSTAYMELSSLRSEDTAV<br>YYCARGWGREQLAPHPSQYYYYYYGMDVWGQ<br>GTTVTVSS	EIVMTQSPGTPSLSPGERATLSCRASQ<br>SIRSTYLAWYQQKPGQAPRLLIYGASS<br>RATGIPDRFSGSGSGTDFTLTISRLEP<br>EDFAVYYCQQYGRSPSFGQGTKVEIK
m102.4	US 2009/0214428 A1 (2009)	EVQ**VI**QSGA**D**VKK**P**GSSVKVSCKSSGGTFS**K**<br>YAINWVRQAPGQGLEWMGGIIPILGIANYAQ<br>KFQGRVTITTDESTSTAYMELSSLRSEDTAV<br>YYCARGWGREQLAPHPSQYYYYYYGMDVWGQ<br>GTTVTVSS	EIVMTQSPGTLSLAPGERATLSCWASQ<br>SVRNNYLAWYQQKPGQAPRLVIYNGST<br>RATGIPDRFSGSGSGTDFTLTISRLDP<br>EDFAVYYCQQYGNSRRVTFGGGTKVEI<br>K
	Xu *et al.*[2]	EVQ**LV**QSGA**E**VKK**R**GSSVKVSCKSSGGTFS**N**<br>YAINWVRQAPGQGLEWMGGIIPILGIANYAQ<br>KFQGRVTITTDESTSTAYMELSSLRSEDTAV<br>YYCARGWGREQLAPHPSQYYYYYYGMDVWGQ<br>GTTVTVSS	EIVMTQSPGTLSLAPGERATLSCWASQ<br>SVRNNYLAWYQQKPGQAPRLVIYNGST<br>RATGIPDRFSGSGSGTDFTLTISRLDP<br>EDFAVYYCQQYGNSRRVTFGGGTKVEI<br>K

References

1. Pires, D. E. & Ascher, D. B. mCSM-AB: a web server for predicting antibody-antigen affinity changes upon mutation with graph-based signatures. *Nucleic Acids Res* **44**, W469-473, doi:10.1093/nar/gkw458 (2016).
2. Xu, K. *et al.* Crystal structure of the Hendra virus attachment G glycoprotein bound to a potent cross-reactive neutralizing human monoclonal antibody. *PLoS Pathog* **9**, e1003684, doi:10.1371/journal.ppat.1003684 (2013).
